# Phage-Derived Depolymerase as an Antibiotic Adjuvant Against Multidrug-Resistant *Acinetobacter baumannii*

**DOI:** 10.3389/fmicb.2022.845500

**Published:** 2022-03-25

**Authors:** Xi Chen, Miao Liu, Pengfei Zhang, Miao Xu, Weihao Yuan, Liming Bian, Yannan Liu, Jiang Xia, Sharon S. Y. Leung

**Affiliations:** ^1^Department of Chemistry, The Chinese University of Hong Kong, Sha Tin, Hong Kong SAR, China; ^2^School of Pharmacy, The Chinese University of Hong Kong, Sha Tin, Hong Kong SAR, China; ^3^Department of Biomedical Engineering, The Chinese University of Hong Kong, Sha Tin, Hong Kong SAR, China; ^4^Emergency Medicine Clinical Research Center, Beijing Chao-Yang Hospital, Capital Medical University, Beijing, China

**Keywords:** depolymerase, exopolysaccharide degrading enzymes, antibiotic adjuvant, serum killing, biofilm prevention and degradation

## Abstract

Bacteriophage-encoded depolymerases are responsible for degrading capsular polysaccharides (CPS), lipopolysaccharides (LPS), and exopolysaccharides (EPS) of the host bacteria during phage invasion. They have been considered as promising antivirulence agents in controlling bacterial infections, including those caused by multidrug-resistant (MDR) bacteria. This feature inspires hope of utilizing these enzymes to disarm the polysaccharide capsules of the bacterial cells, which then strengthens the action of antibiotics. Here we have identified, cloned, and expressed a depolymerase Dpo71 from a bacteriophage specific for the gram-negative bacterium *Acinetobacter baumannii* in a heterologous host *Escherichia coli*. Dpo71 sensitizes the MDR *A. baumannii* to the host immune attack, and also acts as an adjuvant to assist or boost the action of antibiotics, for example colistin. Specifically, Dpo71 at 10 μg/ml enables a complete bacterial eradication by human serum at 50% volume ratio. A mechanistic study shows that the enhanced bactericidal effect of colistin is attributed to the improved outer membrane destabilization capacity and binding rate to bacteria after stripping off the bacterial capsule by Dpo71. Dpo71 inhibits biofilm formation and disrupts the pre-formed biofilm. Combination of Dpo71 could significantly enhance the antibiofilm activity of colistin and improve the survival rate of *A. baumannii* infected *Galleria mellonella*. Dpo71 retains the strain-specificity of the parent phage from which Dpo71 is derived: the phage-sensitive *A. baumannii* strains respond to Dpo71 treatment, whereas the phage-insensitive strains do not. In summary, our work demonstrates the feasibility of using recombinant depolymerases as an antibiotic adjuvant to supplement the development of new antibacterials and to battle against MDR pathogens.

## Introduction

Carbapenem-resistant *Acinetobacter baumannii* was identified as the number one priority pathogen by World Health Organization [WHO] (2017) and Centers for Disease Control and Prevention [CDC] (2019). *A. baumannii* infection is associated with frequent and hard-to-treat infections, such as pneumonia, bacteremia, urinary tract infections, meningitis, and wound infections (Peleg et al., 2008). In the past decades, outbreaks of *A. baumannii* resistant to the last-resort antibiotics, such as colistin, are increasingly reported (Pendleton et al., 2013; Fair and Tor, 2014). Alarmingly, the development of novel antibiotics have experienced significant setbacks in recent years (World Health Organization [WHO], 2019), and thereby the field is urgently calling for novel antibacterial agents to address the clinical challenges of *A. baumannii* associated infections.

Bacteriophages (phages), natural co-evolving bacteria killers, are being revitalized to combat multidrug-resistant (MDR) bacteria (Hampton et al., 2020). Although regarded as a promising alternative to conventional antibiotics, phage therapy faces challenges as very few completed clinical trials confirmed the efficacy (Pirnay and Kutter, 2020). The narrow host range and the development of phage-resistance could be the major factors for such failures. The viral nature of phage may also be unacceptable to most clinicians and the general public (Verbeken et al., 2014a,b, 2016). Alternatively, researchers explore the potential of phage-encoded enzymes, including the peptidoglycan hydrolases, polysaccharide depolymerases, and holins, as novel antibacterial agents, drawing inspiration from the life cycle of phage. In contrast to phage, phage-encoded enzymes, as therapeutic proteins without replicating capability are more manageable and acceptable (Pires et al., 2016; Latka et al., 2017; Knecht et al., 2020; Lai et al., 2020).

Phage-encoded depolymerases are polysaccharide hydrolases or lyases responsible for stripping bacterial polysaccharides, including exopolysaccharides (EPS), capsular polysaccharides (CPS), and lipopolysaccharide (LPS), to facilitate the parent phage to inject its DNA materials into the bacterial host (Pires et al., 2016; Knecht et al., 2020). Distinct from other phage-encoded enzymes, depolymerases do not lyse bacterial cells directly. Instead, they disintegrate the CPS of bacteria to make them susceptible to host immune attack and antibacterial treatments (Majkowska-Skrobek et al., 2018). Recombinant depolymerases have been shown to protect mice from fatal systemic bacterial infections (Lin et al., 2014; Chen et al., 2020) and to disrupt biofilms for enhanced antimicrobial activity (Hughes et al., 1998; Gutiérrez et al., 2015; Hernandez-Morales et al., 2018; Mi et al., 2019; Topka-Bielecka et al., 2021).

Combined administration of depolymerases and antibiotics will produce superior antibacterial efficacy – expected but not yet well-supported by experiments. Bansal et al. (2014) were the first to report the adjuvant effect of a depolymerase derived from *Aeromonas punctata* (a facultative anaerobic gram-negative bacterium) with gentamicin in treating mice infected with non-lethal dose of *K. pneumoniae*. Intranasal administration and intravenous administration of the combination for lung infection and systemic infection, respectively, both reduced bacterial counts significantly more than the single-agent treatments. They attributed the improved bacterial killing efficiency to the enhanced bacterial susceptibility toward gentamicin after the bacteria were decapsulated by the depolymerase. Depolymerases also effectively dispersed the EPS matrix in *K. pneumoniae* biofilms to facilitate the penetration of gentamicin (Bansal et al., 2015). A similar synergy was also observed in the treatment of *K. pneumoniae* biofilms using the Dep42 depolymerase and polymyxin B (Wu et al., 2019). On the contrary, Latka and Drulis-Kawa (2020) showed that the KP34p57 depolymerase had no impact on the activity of ciprofloxacin but could significantly enhance the antibiofilm efficiency of non-depolymerase-producing phages. Depolymerase could also enhance the antibiofilm efficacy of a phage-encoded antibacterial enzyme endolysin (Olsen et al., 2018).

Currently, a few *A. baumannii* depolymerases have been identified (Hernandez-Morales et al., 2018; Liu et al., 2019a; Oliveira et al., 2019a,b; Wang et al., 2020; Shahed-Al-Mahmud et al., 2021). Whether they would work with antibiotics in controlling biofilm-associated infections, like those observed for *K. pneumoniae*, remains questionable. In the present study, the combinational effects of a depolymerase Dpo71 encoded by a lytic *A. baumannii* phage, vB_AbaM-IME-AB2 (IME-AB2 in short) (Peng et al., 2014; Liu et al., 2016), with serum or colistin in targeting MDR *A. baumannii* are evaluated.

## Materials and Methods

### Bacterial Strains, Culture Condition and Materials

All bacterial strains used in this study are listed in Supplementary Table 1. The multidrug-resistant *A. baumannii* strain MDR-AB2, isolated from the sputum samples of a patient with pneumonia at PLA Hospital 307 was supplied by the Beijing Institute of Microbiology and Epidemiology (Peng et al., 2014). The antibiotic susceptibility profile of this strain was determined, which confirmed its resistance to most of the commonly used antibiotics (Peng et al., 2014). All the bacterial strains were grown in Nutrient Broth (NB) medium at 37°C. Colistin was bought from J&K Scientific (Beijing, China) (Cat No. 437689, >19,000 IU/mg).

### Plasmid Construction

The plasmid was constructed using standard cloning methods. Genes encoded Dpo71 (Protein id: YP_009592222.1) was synthesized by BGI (Shenzhen, China) and cloned into the pET28a plasmid using *Bam*HI and *Xho*I site and the protein sequence was listed in supporting information (Supplementary Table 2).

### Recombinant Proteins Expression and Purification

The constructed plasmid was transformed into *Escherichia coli* BL21 (DE3) cells and colonies were grown overnight at 37°C in LB media supplemented with 50 μg/ml kanamycin. The start culture was grown overnight, and then was used to inoculate LB media supplemented with antibiotics at 1:100 ratio. The cell culture was grown at 37°C to reach OD_600_ ∼0.6 before 0.25 mM IPTG was added to induce protein expression. After grown at 16°C overnight, cells were harvested for protein purification. The enzyme was purified by nickel affinity chromatography using HisTrap™ HP column (GE Healthcare, Chicago, United States). Briefly, harvested cells were re-suspended in lysis buffer containing 10 mM imidazole, 50 mM phosphate/300 mM sodium chloride (pH 8.0). The cell suspension was lysed by sonication and centrifuged. The supernatant was collected, filtered, and loaded into the column. The bound protein was eluted by imidazole gradient from 10 to 500 mM. Pure protein fractions eluted with imidazole gradient were collected and exchanged with PBS (pH 7.4). After purification, all proteins were flash frozen under liquid nitrogen and stored at −80°C. Protein concentration was determined using NanoDrop (Thermo Fisher Scientific, Massachusetts, United States).

### Size Exclusion Chromatography Analysis

Purified depolymerase Dpo71 as well as two gel filtration markers with molecular weight of 150 and 200 kDa (MWGF200, Sigma-Aldrich, Missouri, United States) were used for analysis, performed on an AKTA FPLC instrument installed with a Superose 6 Increase prepacked column (GE Healthcare, Illinois, United States). Proteins were filtered with 0.2 μm membrane and injected into the FPLC system equilibrated with PBS.

### Circular Dichroism Spectroscopy

Far-UV CD spectroscopy is commonly used to analyze the secondary structures of proteins with a Jasco J810 CD spectrometer (Oliveira et al., 2020). The spectrum measurement was performed with the Dpo71 of 0.30 mg/ml in PBS buffer (pH 7.4) using a wavelength range from 190 to 260 nm. Thermal denaturation with 1°C/min increments was also employed to measure the secondary structure unfolding at 215 nm, from 20 to 90°C. The melting curves were fitted into a Boltzmann sigmoidal function.

### Spot Test Assay

The depolymerase activity of Dpo71 was qualitatively assayed by a modified single-spot assay. In brief, 100 μl of MDR-AB2 overnight bacterial culture was added to 5 ml of molten soft nutrient agar (0.7%) and incubated at 37°C for 3 h to form a bacterial lawn in plates. The purified enzyme was serially diluted, then 5 μl of each dilution (from 0.001 to 10 μg) was dropped onto the MDR-AB2 bacterial lawn for incubation at 37°C overnight. The plates were monitored for the formation of semi-clear spots as a confirmation of the depolymerase activity.

### Extraction of Bacterial Surface Polysaccharides

The bacterial polysaccharide extracts (containing both CPS and LPS) were purified, via a modified hot water-phenol method as described previously (Liu et al., 2020). Briefly, *A. baumannii* were cultured overnight in LB with 0.25% glucose. A volume of 1 ml culture was centrifuged (10,000 rpm, 5 min) and resuspended in 200 μl of double distilled water (ddH_2_O). An equal volume of water-saturated phenol (pH 6.6; Thermo Fisher Scientific) was added to the bacterial suspension. The mixture was vortexed and incubated at 65°C for 20 min, centrifuged at 10,000 rpm for 10 min. Then the supernatant was extracted with chloroform to remove bacterial debris. The obtained bacterial CPS/LPS were lyophilized and stored at –20°C before use.

### Quantification of Depolymerase Activity

The enzymatic activity of Dpo71 degrading the bacterial polysaccharides was determined as described in Liu et al. (2020) with minor modifications. The extracted CPS/LPS powder of *A. baumannii* was resuspended in ddH2O (1 mg/ml) and mixed with Dpo71 (30 μg/ml) or deactivated Dpo71 (by heating at 90°C for 15 min) to a final reaction volume of 200 μl. The extracted CPS/LPS or enzyme alone served as the controls. After 2 h incubation at 37°C, cetylpyridinium chloride (CPC, Sigma-Aldrich, Missouri, United States) was added to the mixture at the final concentration of 5 mg/ml, which was further incubated at room temperature for 5 min. Absorbance was measured at 600 nm using a microplate reader (Multiskan Sky, Thermo Fisher Scientific, Massachusetts, United States). The experiment was performed in triplicate and repeated at least in two independent experiments.

### Influence of pH and Storage Time on the Depolymerase Activity

The extracted CPS/LPS powder was dissolved in 100 mM citric acid-Na_2_HPO_4_ buffer (pH 3.0–8.0) or 100 mM Glycine-NaOH buffer (pH 9.0–10.0) to a final concentration of 1 mg/ml. The CPS/LPS solutions of *A. baumannii* were mixed with Dpo71 (30 μg/ml) to a final reaction volume of 200 μl, respectively. After 2 h incubation at 37°C, the turbidity of residual CPS/LPS in various pH buffers was determined as described above. The effect of pH on the enzymatic activity was determined by this method. The storage stability of Dpo71 at 4°C was determined by measuring the CPS/LPS degradation activity after 1, 3, and 6 months storage. All assays were performed in triplicate and repeated at least in two independent experiments.

### Serum Killing Assay

The serum killing assay was performed as previously described (Oliveira et al., 2020). Log phase bacteria were prepared by inoculating overnight culture at 1:100 ratio in NB medium and shaking 180 rpm for 3–4 h at 37°C. Then the cells of each culture (AB#1, AB#2, AB#3, and AB#4) were harvested via centrifugation, washed, and resuspended in PBS, then adjusted to OD_600_ = 0.6. Human serum (Sigma-Aldrich, Missouri, United States) was mixed with (i) only bacteria or (ii) bacteria and Dpo71 mixture. The Dpo71 was fixed at final concentration of 10 μg/ml and the volume ratio of human serum was set at 50%. Experiments with heat-inactivated serum (at 56°C for 30 min) were served as controls. The mixtures were then incubated at 37°C for 4 h for viable bacterial counting. Time-killing assays were also performed for the two sensitive strains (AB#1, AB#2) with the volume ratio of human serum varied from 1 to 50% and at 100 μg/ml Dpo71. Samples were withdrawn at 1, 3, 5, and 24 h for bacterial counting. All assays were performed in triplicate and repeated at least in two independent experiments.

### Scanning Electron Microscopy

Scanning Electron Microscopy (SEM) was conducted as described previously (Oliveira et al., 2019a). Log phase *A. baumannii* bacteria were washed twice with PBS and resuspended in PBS buffer at OD_600_ = 0.6. Then approximately 10^8^ cells were incubated at 37°C with PBS or Dpo71 for 3 h. Cells were then fixed with 2.5% (v/v) glutaraldehyde at 4°C overnight. Thereafter, the fixed cells were washed twice with PBS and dehydrated with a graded ethanol series (15, 30, 50, 70, 85, and 100% for twice). The bacterial suspensions were then spotted on glass and dried with vacuum. Finally, the samples were coated with gold and observed with Quanta 400F SEM (FEI, Oregon, United States).

### Outer Membrane Permeability to 1-N-Phenylnaphthylamine Assay

For the investigation of outer membrane permeability, 1-N-phenylnaphthylamine (NPN) uptake assay was performed (Helander and Mattila-Sandholm, 2000; Chen et al., 2021). *A. baumannii* cells were grown to mid-log phase (OD_600_ = 0.6–0.8), centrifuged, and resuspended in PBS. Then PBS (control), Dpo71 (10 μg/ml), colistin (1 μg/ml) or their combination (Dpo71 + colistin) were incubated with 10^7^ CFU/ml cells in the presence of 10 μM NPN for 5 min. The fluorescence intensities were recorded using a microplate reader (CLARIOstar, BMG Labtech, Ortenberg, Germany) with 350 ± 7.5 nm for excitation and 420 ± 10 nm for emission. All experiments were performed in three biological replicates and repeated at least in two independent times.

### Assessment of Colistin Binding

The assay described by Campos et al. (2004) was done with minor modifications. Briefly, cells grown as described above were resuspended in PBS buffer about 10^10^ CFU/ml. Then, the bacteria were first treated with Dpo71 or PBS at 37°C for 2 h. Then 50 μg/ml colistin was added, after 5 min of incubation, the cells with the bound colistin were sedimented (12,000 g, 10 min), the supernatant was centrifuged two more times under the same conditions. Finally, the unbound colistin was quantified with HPLC. The integrated areas were recorded and the concentrations of the unbound colistin were calculated from the standard curve of colistin (16–500 μg/ml). All experiments were performed in three biological replicates and repeated at least in two independent times.

### Biofilm Inhibition Assay

*A. baumannii* were grown in NB medium overnight at 37°C with continuous shaking 180 rpm. The overnight bacterial culture was diluted with fresh NB medium to a final density of 10^6^ CFU/ml. Then the diluted bacteria cultures were treated with PBS (control), Dpo71 (1, 10, or 40 μg/ml), colistin (1 μg/ml) or their combination (Dpo71 + colistin) with a final volume of 150 μl/well at 37°C for 24 h with gentle shaking (100 rpm). At the end of the incubation time, all media were removed and the wells were stained with 200 μl 0.1% (w/v) crystal violet for 1 h. After staining, the crystal violet solution was removed and the wells were washed with 200 μl PBS for three times. Then, 200 μl of 70% ethanol was added to dissolve the crystal violet and 100 μl solution was transferred to a new plate for quantification of the residual biofilm biomass using a microplate reader (CLARIOstar, BMG Labtech, Ortenberg, Germany) at 570 nm. All experiments were performed in three biological replicates and repeated at least in two independent times.

### Biofilm Removal Assay

*A. baumannii* were grown in NB medium overnight at 37°C with continuous shaking 100 rpm. The overnight bacterial culture was diluted with fresh NB medium to a final density of OD_600_ = 0.2. To initiate the biofilm growth, diluted culture was aliquoted into a 96-well plate at 100 μl/well (Costar, Corning Incorporated, New York, United States) and incubated at 37°C for 24 h at 100 rpm. Biofilm was washed twice with PBS and treated with PBS (control), Dpo71 (10 μg/ml), colistin (4 μg/ml) or their combination (Dpo71 + colistin) with a final volume of 150 μl/well at 37°C for 24 h with gentle shaking (100 rpm). At the end of the incubation time, the residual biomass was quantified using crystal violet as described in the inhibition assay above. The antibiofilm activity was also evaluated by the counting the viable bacteria in the biofilm (Wilson et al., 2017). Briefly, the biofilm was grown and treated as above, then the wells were washed three times with PBS. Then the biofilm-containing wells were mixed fully with a pipetting device, making the biofilm cells become planktonic cells. Each sample was serially diluted and plated for bacterial counting. All experiments were performed in triplicate and repeated at least in two independent times.

### Confocal Laser Scanning Microscopy on Biofilm Removal

Confocal dish (NEST, Wuxi NEST Biotechnology, Jiangsu, China) was used instead of the 96-well plate for the antibiofilm study described above. Before microimaging, the biofilms were stained by LIVE/DEAD™ BacLight™ Bacterial Viability Kit (Thermo Fisher Scientific, Massachusetts, United States) for 60 min following the manufacturer’s instructions. Then the biofilms were washed three times with PBS before the confocal laser scanning microscopy (Nikon, C2+ confocal, Tokyo, Japan) study. The excitation maximum and emission maximum of SYTO 9 is at 483 and 503 nm, respectively. The excitation maximum and emission maximum of propidium iodide is at 493 and 636 nm, respectively.

### Hemolysis Assay

The effect of Dpo71 on the hemolysis of human red blood cells was performed using previously described methods with minor modifications (Liu et al., 2019a). The human blood sample from a healthy donor was centrifuged at 1,000 rpm for 10 min to remove the serum. The red blood cell pellets were washed with PBS (pH = 7.4) at least three times and then diluted to a concentration of 5% (volume ratio) with PBS. The Dpo71 (10, 100, and 500 μg/ml, final concentration) was added to the red blood cells and incubated at 37°C for 1 h, followed by centrifugation at 1,000 rpm for 10 min. Then 100 μl supernatant was transferred to a 96-well microplate and topped up with another 100 μl of PBS to get a final volume of 200 μl. The erythrocytes in PBS and 0.1% Triton X-100 were served as negative and positive controls, respectively. The hemoglobin in supernatant was determined by measuring absorbance at 540 nm using a microplate reader (Multiskan Sky, Thermo Fisher Scientific, Massachusetts, United States). All experiments were performed in three biological replicates and repeated at least in two independent times.

### Cytotoxicity of Dpo71 Against BEAS-2B Cell

BEAS-2B (Human Normal Lung Epithelial Cells) cells were cultured in DMEM (GIBCO, New York, United States) containing 10% FBS (GIBCO, New York, United States) under standard conditions in a humidified incubator with 5% CO_2_ at 37°C. The cytotoxic effect of the Dpo71 on BEAS-2B cells was measured by Cell Counting Kit-8. The BEAS-2B cells were seeded at density of 10^4^ cells/well in a 96-well plate containing 200 μl of culture medium and incubated at 37°C for 24 h. Next, the cells were incubated with Dpo71 for 12 h followed by incubating with 10 μl of WST-8 solution (Beyotime, Shanghai, China) for another 2 h at 37°C. Absorbance was measured at a wavelength of 450 nm using a microplate reader (Multiskan Sky, Thermo Fisher Scientific, Massachusetts, United States). The PBS group was served as a negative control. All experiments were performed in three biological replicates and repeated at least in two independent times.

### *Galleria mellonella* Infection Model

The *G. mellonella* model was conducted following the procedures described by Peleg et al. (2009) with some minor modifications, and referring in other *G. mellonella* studies (Yang et al., 2015; Blasco et al., 2020). The *G. mellonella* larvae were acquired from WAGA company in Hong Kong and the injection was performed with a 10 μl SGE syringe (Sigma-Aldrich, Missouri, United States). To infect the *G. mellonella*, the larvae were first injected with 10^6^ CFU *A. baumannii* (MDR-AB2 strain) into the last left proleg. Then the PBS buffer (control group) or 5 μg Dpo71; 1 μg colistin; or 1 μg colistin + 5 μg Dpo71 (treatment group), were injected into the last right proleg within 30 min. The *G. mellonella* were then incubated at 37°C and observed at 24 h intervals over 4 days. The *G. mellonella* which did not respond to physical stimuli were considered dead. Each group included nine *G. mellonella* with individual experiments repeated two times (*n* = 18).

### Statistics

All experimental data are presented as means ± standard deviation (SD), and significance was determined using independent Student’s *t* tests and the one-way analysis of variance (ANOVA), assuming equal variance at a significance level of 0.05. Comparison of the survival rates of *G. mellonella* between groups was determined by Kaplan–Meier survival analysis with a log-rank test. All statistical analysis was performed using GraphPad Prism software.

## Results

### Identification and Characterization Depolymerase Dpo71

**FIGURE 1:**
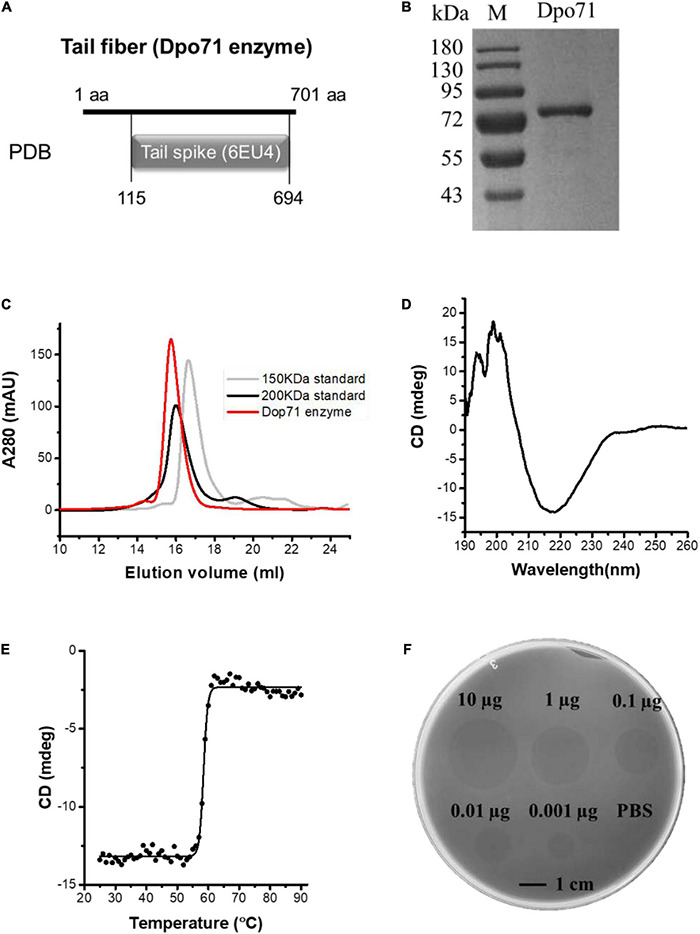
Identification and characterization of Dpo71 depolymerase. **(A)** Bioinformatic analysis indicates the gp71 gene of the IME-AB2 phage. **(B)** SDS gel electrophoresis analysis of purified Dpo71 and a standard molecular mass marker (Lane M). **(C)** Size exclusion chromatography of purified depolymerase protein. **(D)** Circular dichroism analysis of Dpo71 measured in the far-UV (190–260 nm). **(E)** The melting curve of Dpo71 acquired at 215 nm from 20 to 90°C. **(F)** Spot test assay of Dpo71 against *A. baumannii* lawn (0.001–10 μg).

The IME-AB2 phage exhibits plaque surrounded by a halo-zone, suggesting the presence of a depolymerase protein. Bioinformatic analysis reveals that gp71 is a tail fiber protein (Peng et al., 2014) with 43% sequence similarity as the depolymerase encoded by another *Acinetobacter* phage vB_AbaP_AS12 (Protein Data Bank number 6EU4) (Figure 1A). Expression of the ORF71 sequence in *E. coli.* yields a protein with more than 95% purity and a molecular mass of about 80 kDa (Figure 1B), matching the calculated value of 80.2 kDa. Size exclusion chromatography shows that the purified Dpo71 elutes as a single peak at a molecular weight larger than 200 kDa (Figure 1C). This corresponds to a trimer, consistent with the expected oligomeric form of phage tail fiber protein which is believed to endure extreme conditions for phage infection and survival (Oliveira et al., 2019a,^2020^). Circular dichroism (CD) reveals that the Dpo71 protein adopts a well-folded conformation rich in β-sheet structures with a negative dichroic minimum at 215-nm and a positive maximum around 195-nm characteristic peaks (Figure 1D). The melting curves following the CD signal at 215 nm show a melting temperature (Tm) of 58.5°C (Figure 1E). Spot tests were performed next to confirm the ability of Dpo71 in degrading bacterial capsules of the host bacteria MDR-AB2 of the parent phage. Semi-clear spot formation was observed on the bacterial lawn with the spot sizes increasing with the dose of depolymerase from 0.001 to 10 μg range (Figure 1F). The bacterial CPS/LPS degradation activity of Dpo71 was also confirmed by the reduced CPS/LPS concentration and Alcian blue staining (Supplementary Figure 1) using protocols documented in previous studies (Liu et al., 2020).

### Robustness of Dpo71 Upon Administration and Storage

**FIGURE 2:**
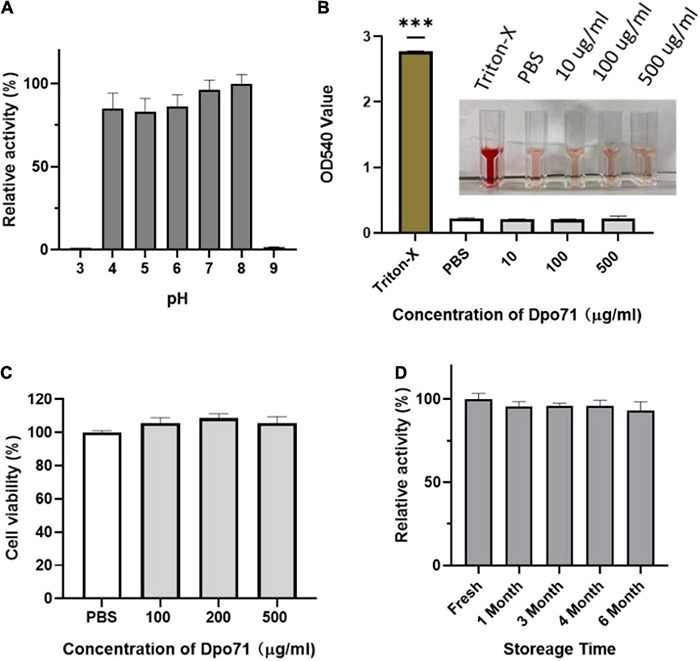
Stability and toxicity of Dpo71. **(A)** The effect of pH on Dpo71 depolymerase activity. CPC turbidity assay was used to measure the polysaccharide extracts (CPS/LPS) degradation activity, the highest activity at pH 8 was set as 100%. **(B)** Hemolysis of red blood cells by Dpo71. PBS and 0.1% Triton X-100 in PBS as the negative and positive controls, respectively. ^***^ indicated *P* < 0.001, Student’s *t*-test. **(C)** Cytotoxicity of Dpo71 against human cells (BEAS-2B). **(D)** Stability of Dpo71 after storage at 4°C. The polysaccharide extract (CPS/LPS) degradation activity of the freshly prepared enzyme was set as 100%. Data are expressed as means ± SD (*n* = 5).

To evaluate the therapeutic applicability of Dpo71, the enzymatic activity of Dpo71 at various pH values was evaluated by monitoring the turbidity of the residual polysaccharide extracts (CPS/LPS). Figure 2A shows that the enzyme remained active in the range of pH 4–8, covering most of the physiological conditions. Then we measured the toxicity of Dpo71 to mammalian cells, human red blood cells and lung bronchial epithelial cell line (BEAS-2B cells). No hemolytic activity was detected even at a high dose of 500 μg/ml (Figure 2B). Figure 2C also shows Dpo71 has no cytotoxicity against BEAS-2B cells. The result suggests that Dpo71 may be a safe treatment, likely for systemic or pulmonary infections. As stability upon storage is critical for the development of commercially viable protein therapeutics, the storage stability of Dpo71 at 4°C has been evaluated. Results show that Dpo71 is stable for at least 6 months without noticeable activity loss (Figure 2D).

### Sensitizing Bacteria to Serum Killing and Antibiotic

**FIGURE 3:**
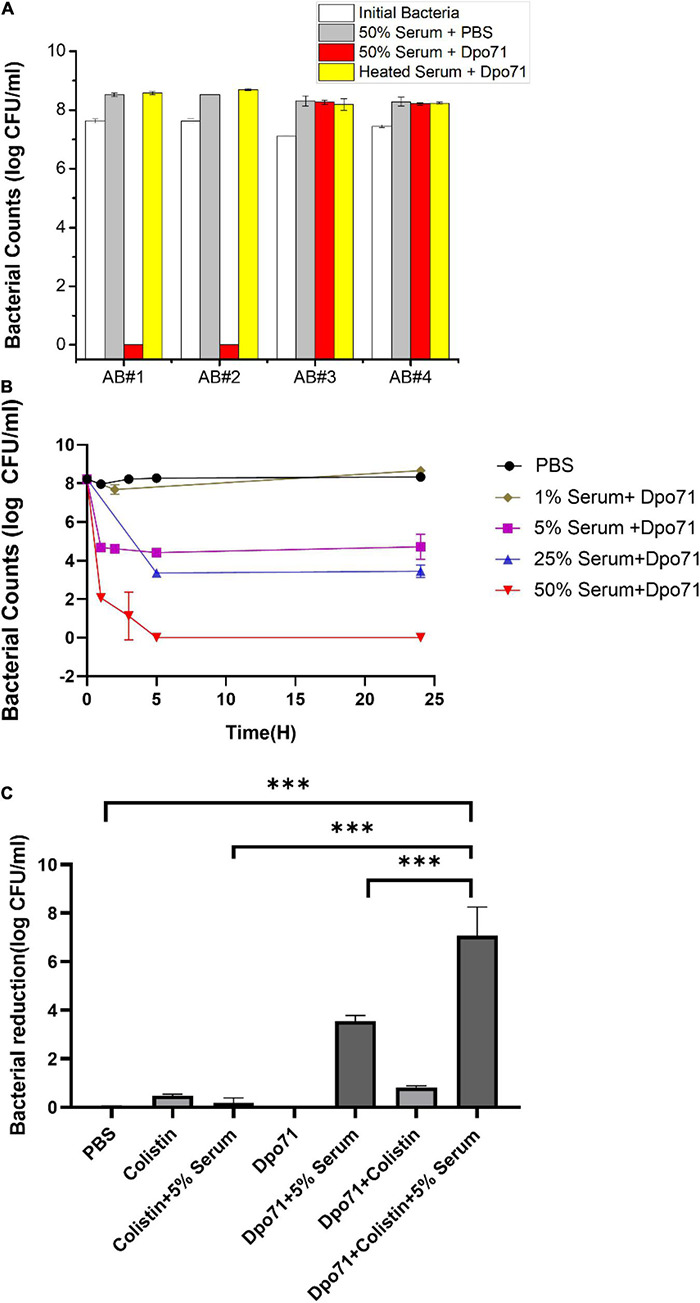
Dpo71 enhanced the serum sensitivity and colistin activity against *A. baumannii.* **(A)** Bacterial susceptibility to the treatment with Dpo71 concentration of 10 μg/ml and 50% serum volume ratio. *A. baumannii* clinical strains sensitive (AB#1 and AB#2) and insensitive (AB#3 and AB#4) to the phage IME-AB2 were tested. **(B)** Time-killing curve of Dpo71 (10 μg/ml) against MDR-AB2 (AB#2 strain) in the presence of 1–50% human serum. **(C)** Dpo71 and colistin activity against *A. baumannii* in the presence/absence of 5% human serum. Data are expressed as means ± SD (*n* = 3). ^***^ indicates *P* < 0.001, Student’s *t*-test.

As depolymerase can disintegrate bacterial capsules and thus sensitize bacteria to be killed by host immune system (Yoshida et al., 2000; Spinosa et al., 2007; Oliveira et al., 2020), we first measured whether serum could kill the Dpo71-treated bacterial cells. Two *A. baumannii* strains (AB#1 and AB#2), belonging to ST208 sequence type (Liu et al., 2019a), sensitive to the parent IME-AB2 phage and two insensitive strains (AB#3 and AB#4) were chosen for the serum killing assays. The four tested strains were resistant to serum killing and continue to grow in human serum without the presence of depolymerase. Bacteria treated with 10 μg/ml Dpo71 and inactivated serum also showed no antibacterial effect (Figure 3A). On the contrary, a remarkable bacterial reduction (8 log) was noted for the two sensitive strains (AB#1 and AB#2), but not for the insensitive ones (AB#3 and AB#4) when the bacteria were treated with active human serum (with a volume ratio of 50%) and Dpo71 (Figure 3A). Furthermore, the time-killing assay on the two sensitive strains was performed with a serum ratio of 1–50%. Complete bacterial eradication was observed after a 5-h treatment at 50% human serum for both AB#1 and AB#2 (Figure 3B and Supplementary Figure 2). It is noteworthy that a 5% serum was sufficient to achieve around 4-log bacterial reduction after 5 h and with minor regrowth after 24 h. This efficacy is significantly higher as compared with previous reports, in which at least 25% human serum is needed to achieve the same killing efficiency (Majkowska-Skrobek et al., 2018; Liu et al., 2019a,^2020^; Mi et al., 2019; Oliveira et al., 2020).

We next examined the antibiotic adjuvant effect of Dpo71 against MDR-AB2. Colistin was chosen because it was the only tested antibiotic that the MDR-AB2 strain was susceptible to with a minimum inhibition concentration (MIC) of 2 μg/ml (Peng et al., 2014; Supplementary Table 3). The colistin concentration was set at 1 μg/ml, half of the MIC value. Figure 3C shows that 1 μg/ml colistin alone or colistin with 5% serum had no antibacterial effect against the inoculation of 10^8^ CFU/ml. Dpo71 combined with 5% serum could achieve around 4-log bacterial reduction and the antibacterial effect was further enhanced to nearly complete eradiation (residual viable bacteria reduced from 4.4 ± 0.2 log to 0.7 ± 1.1 log) when Dpo71 was used in combination with 1 μg/ml colistin in the presence of 5% serum (Figure 3C). This boosting effect was consistent with a modified checkerboard assay examining the synergy between Dpo71 and colistin that the MIC of colistin dropped from 2 to 0.5 μg/ml with the addition of 5% serum (Data not shown). Notably, these results indicated that Dpo71 could act as an adjuvant to boost the antibacterial activity of colistin in low serum condition.

### Mechanisms for the Adjuvant Effect of Depolymerase on Colistin

**FIGURE 4:**
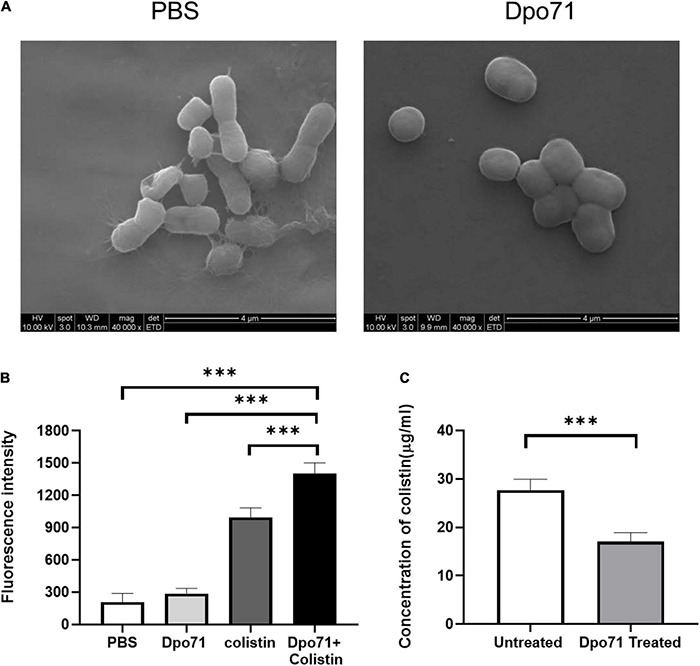
Mechanistic studies using the Dpo71 as an adjuvant agent. **(A)** Representative Scanning Electron Microscope (SEM) images of *A. baumannii* incubated with PBS and Dpo71 (10 μg/ml). Scale bar 4 μm. **(B)** NPN uptake assay of *A. baumannii* induced by PBS buffer; Dpo71; colistin (1 μg/ml); and their combination agents (Dpo71 + colistin). Net fluorescence signals with the background signal of the cells subtracted are used. Data are expressed as means ± SD (*n* = 3) with ^***^p < 0.001 determined by Student’s *t*-test. **(C)** Assessment concentration of the unbounded colistin. *A. baumannii* were treated with Dpo71 or PBS for 2 h, then after 5 min incubation of the colistin, the cells with the bound colistin were sedimented. The supernatant was centrifuged and the unbound colistin was measured with the HPLC. Data are expressed as means ± SD (*n* = 3) with ^***^p < 0.001 determined by Student’s *t*-test.

Although the combined administration of depolymerase and antibiotics was superior to the individual treatments, the underlying mechanisms responsible for the adjuvant effect of Dpo71 have not been fully investigated (Bansal et al., 2014, 2015; Olsen et al., 2018; Wu et al., 2019; Latka and Drulis-Kawa, 2020). We first confirmed that Dpo71 could strip the bacterial capsule using scanning electron microscopy (SEM). Figure 4A shows clearly the morphological changes of the bacterial surface after Dpo71 treatment. The bacterial surface of untreated bacteria possessed a complex polysaccharide capsule with pilus-shaped protrusions, while the surface of the Dpo71-treated group showed the absence of these pilus-shaped protrusions, suggesting the loss of the capsule. Then we evaluated the impact of the capsule on the outer membrane (OM) destabilization capacity of colistin using the 1-N-phenylnaphthylamine (NPN) uptake assay. The fluorescence intensity of NPN for the Dpo71-alone group was comparable with the control group and that of the Dpo71-colistin treated bacteria (decapsulated) was significantly higher than the colistin-alone groups (Figure 4B). These results indicate that Dpo71 depolymerase only acted on bacterial capsules and the removal of the capsule could significantly enhance the OM destabilization capability of colistin. The binding efficiency of colistin with native and decapsulated bacteria was estimated. The Dpo71 treated bacteria had a lower level of free colistin in the supernatant compared with the untreated group, indicating that more colistin binds onto the capsule-stripped bacteria (Figure 4C). These results all suggested that depolymerase can function as an antibiotic adjuvant by disintegrating the bacteria capsule to promote interaction between antibiotics and the bacteria, and hence their entry to the bacterial host.

### Anti-biofilm Activity

**FIGURE 5:**
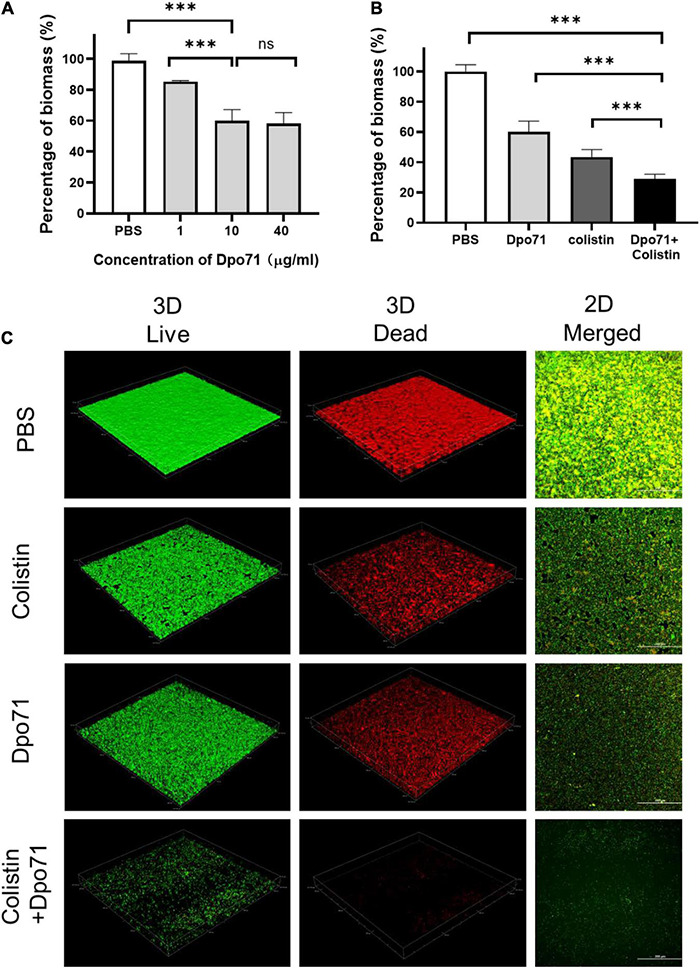
Dpo71 and colistin inhibited biofilm formation. **(A)** Dpo71-alone inhibited the biofilm in a dose-dependent manner. **(B)** Dpo71 and colistin combination inhibited the biofilm formation. *A. baumannii* were incubated with PBS buffer; Dpo71; colistin (1 μg/ml); and their combination agents (Dpo71 + colistin) in 96-well plates for 24 h, followed with crystal violet staining. The OD value of the PBS control was 1.55 ± 0.11. Data are expressed as means ± SD (*n* = 3) with ^***^p < 0.001 determined by Student’s *t*-test. **(C)** The Representative confocal fluorescence microscopic images of LIVE/DEAD stained *A. baumannii* biofilm (Scale bar, 200 μm). ns: No significant difference.

We next measured the inhibition effect of Dpo71 on biofilm formation (Wilson et al., 2017). Figure 5A shows Dpo71 inhibits biofilm formation in a dose-dependent manner. At 1 μg/ml Dpo71, the residual biomass was 80% as compared with the PBS-treated control. The residual biomass was further reduced to 60.0 ± 7.2% at 10 μg/ml Dpo71 and 58.2 ± 7.0% at 40 μg/ml. Therefore, 10 μg/ml Dpo71 was chosen for the evaluation of adjuvant effects with 1 μg/ml colistin (1/2 MIC) in inhibiting biofilm formation. Colistin alone brought down the biomass to 43.5 ± 4.9%, and further reduced it to 28.9 ± 3.1% when used in combination with Dpo71 (Figure 5B). The biofilm was visualized by the LIVE/DEAD staining, in which live cells were stained with green fluorescence and dead cells with damaged membrane were stained red (Figure 5C). Consistent with the results from the CV staining assay, confocal imaging showed Dpo71 alone and colistin alone could prevent the biofilm formation to a certain extent compared with the PBS-treated sample, but the combination of Dpo71 and colistin yielded the most effective biofilm inhibition. Our results confirmed the capability of both Dpo71-alone and co-treatment with colistin in preventing *A. baumannii* biofilm formation.

**FIGURE 6:**
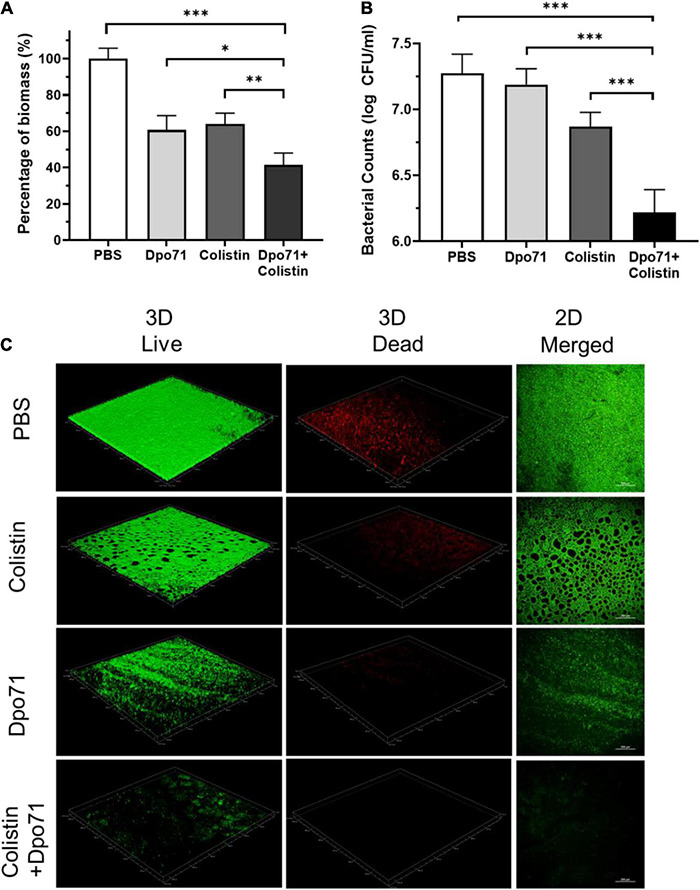
Dpo71 and colistin disrupted the pre-formed biofilm. The residual biofilm was assessed by **(A)** crystal violet staining (the OD value of the PBS control was 2.12 ± 0.14) and **(B)** *A. baumannii* bacterial counts. Briefly *A. baumannii s*train was grown on 96-well plates for 24 h for biofilm formation first, and then the biofilm was treated with PBS buffer, Dpo71 (10 μg/ml), colistin (4 μg/ml) or Dpo71 + colistin for 24 h, followed with crystal violet staining and bacterial counting. Data are expressed as means ± SD (*n* = 3). *p < 0.05, ^**^p < 0.01, ^***^p < 0.001, Student’s *t*-test. **(C)** Representative confocal fluorescence microscopic images of live/dead stained *A. baumannii* biofilm (Scale bar, 200 μm). Confocal dish was used instead of the 96-well plate here.

We next assessed whether Dpo71 or its combination with colistin can remove pre-formed biofilm (Wilson et al., 2017). According to the CV staining assay, the biomass could be disrupted by a single treatment of Dpo71 (10 μg/ml) or colistin (4 μg/ml, 2× MIC) to around 60% residual biomass of the PBS control (Figure 6A). The combined treatment could further reduce the residual biomass to 41.5 ± 6.6%. We reached the same conclusion when we compared the number of viable bacterial cells in the dispersed biofilms (Figure 6B). In the absence of Dpo71, colistin was inefficient in killing bacteria embedded in the biofilm (<0.5 log reduction), whereas with the help of Dpo71, more than 90% of the bacterial cells in biofilm were killed (from 7.3 ± 0.1 to 6.2 ± 0.2 in log scale). In the LIVE/DEAD viability assay, the pre-formed biofilm network was dismantled by Dpo71 (Figure 6C). These results show that Dpo71 can efficiently disrupt per-formed biofilm. For biofilms treated with the combination of Dpo71 and colistin, both 2D and 3D confocal images showed only weak fluorescence signals, confirming that the biofilm was effectively removed (Figure 6C). These data indicate that Dpo71 could boost the antibiofilm activity of colistin.

### Antibacterial Activity in a *Galleria mellonella* Infection Model

**FIGURE 7:**
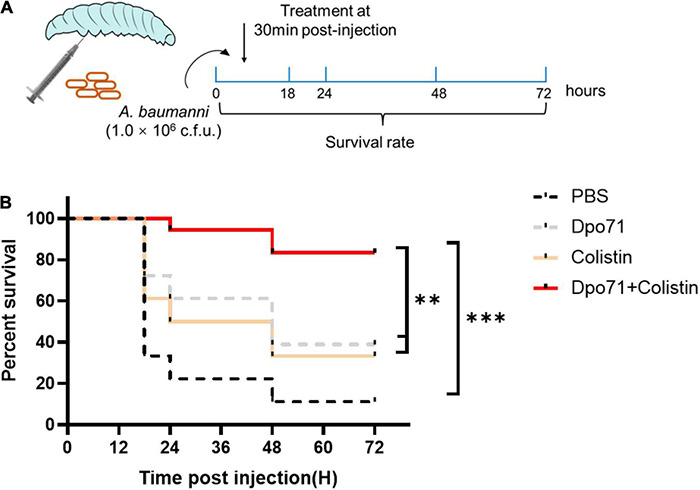
Antivirulent activity in the *Galleria mellonella* infection model. **(A)** Scheme of the experimental protocol for the *G. mellonella.* **(B)** Survival curves for *G. mellonella* infected with 10^6^ CFU *A. baumannii* then followed by the injection of PBS buffer (control group); 5 μg Dpo71; 1 μg colistin; or 1 μg colistin + 5 μg Dpo71 (treatment group) (*n* = 18, ^**^p < 0.01, ^***^p < 0.001, Kaplan–Meier survival analysis with log-rank test).

We next evaluated the *in vivo* efficacy of Dpo71 and colistin in combating bacterial infections in a *Galleria mellonella* infection model (Figure 7A). In the control group, approximately 70% of the *G. mellonella* died within 18 h and the death rate increased to 90% at 48 h (Figure 7B). The survival rate of the colistin-treated group increased to 50% after 24 h post-infection, and around 30% endured to the end of the monitoring period (72 h post-infection). Although depolymerase itself is not bactericidal, the Dpo71-alone treatment was found to be effective in rescuing the infected worms with 40% of the *G. mellonella* surviving for 72 h. The combination treatment increased the survival rate of the infected worms to 80% till the end of the monitoring period, significantly higher than the monotherapy groups (^**^p < 0.01, log-rank test).

## Discussion

*A. baumannii*, one of the most alarming nosocomial gram-negative pathogens, has drawn significant attention in clinical settings due to its exceptional ability to acquire resistance to the commonly used antibiotics (Wieland et al., 2018). Although the knowledge on the mechanisms involved in the pathogenicity of *A. baumannii* is still limited, the production of bacterial capsular polysaccharides has been regarded as an important virulence factor, conferring its intrinsic resistance to peptide antibiotics and protecting it from host immune attack (Wilson et al., 2002; Geisinger and Isberg, 2015; Pettis and Mukerji, 2020). *A. baumannii* has previously shown a much higher biofilm formation rate (80∼91%) compared with other species (5–24%) (Martí et al., 2011). Its excellent capability of forming biofilms also contributes to the bacterial pathogenicity and resistance toward antibiotics. As CPS and EPS (a major component of the biofilms) are the substrates of phage-encoded depolymerases, the application of recombinant depolymerases has received compelling interest as novel antivirulence agents to control multidrug-resistant infections (Pires et al., 2016; Knecht et al., 2020). A few depolymerases encoded by *A. baumannii* phage have been identified in recent years with demonstrated *in vivo* efficacy (Lin et al., 2017; Liu et al., 2019b; Oliveira et al., 2019b; Wang et al., 2020; Shahed-Al-Mahmud et al., 2021). However, the adjuvant effect of depolymerases on SOC antibiotics in controlling infections caused by *A. baumannii* has never been attempted.

In this study, depolymerase Dpo71, derived from an *A. baumannii* phage, IME-AB2 was found to remain active at a pH range of 4-8 and have a Tm of 58.5°C, suggesting it can be used under most physiological conditions. These properties of Dpo71 were consistent with that of other depolymerases (Oliveira et al., 2019a,^2020^). Importantly, this study demonstrated the excellent storage stability of a depolymerase with no noticeable activity loss for at least 6 months storing at 4°C. This would offer great advantages in developing depolymerases as commercially viable antibacterial agents. Depolymerases are known to be highly specific to the capsular type of the bacteria (Whitfield, 2006; Schmid et al., 2015; Singh et al., 2018, 2019). Dpo71 was sensitive to AB#1 and AB#2 but insensitive to AB#3 and AB#4. The reason for this discrepancy is rooted in the mechanism of action of Dpo71, which still remains elusive. The remarkable diversity of bacterial capsules and the narrow host-spectrum of phage and phage-derived enzymes might raise a concern about the general applicability of phage therapy and therapies based on phage-derived enzymes, although in some scenarios this narrow spectrum can be harnessed to eradicate pathogens while leaving beneficial bacterial strains in the flora unharmed. Furthermore, the recombinant Dpo71 effectively decapsulates the host bacteria of its parent phage (AB#1 and AB#2) and re-sensitizes them to serum killing in a serum ratio-dependent manner (Figure 3B). The depolymerase treatment was largely limited for systemic infections because bacterial killing required the aid from the host immune attack such as complement-mediate killing. In the present study, the Dpo71 treated bacteria were significantly reduced in the presence of 5% serum (4 logs of killing from a density of 10^8^ CFU/ml), representing the possibility of applying this depolymerase beyond systemic infection to environments with a low serum level, like lung infections. When the serum ratio increased to 50%, complete bacterial eradication was achieved with Dpo71. This was significantly higher than that reported in previous studies (Lin et al., 2017; Majkowska-Skrobek et al., 2018; Liu et al., 2019a,^2020^; Mi et al., 2019; Oliveira et al., 2020), in which a 50% serum (complement) ratio could only kill 2-5 log of the depolymerase treated bacteria and no further killing was noted when the serum (complement) ratio increased to 75%. Liu et al. (2019a) postulated that the incomplete bacteria-killing was due to the emergence of resistant phenotypes. Therefore, resistance development toward the depolymerase treatment was assessed and compared with the parent phage treatment. While the bacteria developed phage resistance after 24-h co-incubation, they remained sensitive to the Dpo71 depolymerase (Supplementary Table 4). The sensitivity of the bacteria incubating with Dpo71 and 5% serum was also examined. Dpo71 could still yield a clear halo spot on the treated bacteria lawn (Supplementary Figure 3), suggesting they were still sensitive to the depolymerase. It is mainly because depolymerases do not directly kill the bacteria during the antibacterial treatment, reducing the impetus for bacteria to evolve mechanisms against the depolymerases.

While the Dpo71 treatment could effectively remove the bacterial capsules and promoted their interactions with colistin (Figure 4), no enhanced antibacterial effect was noted for the mixture of Dpo71 and colistin in the absence of serum. However, the addition of a small amount of serum (5% volume ratio) significantly boosted the bacterial killing efficiency (Figure 3C). These results suggested that serum was also essential for the combination treatments. Colistin first binds to the LPS at the cell surface followed by displacing the divalent cations (Ca^2+^ and Mg^2+^) to disturb the integrity of the outer membrane, resulting in bacterial cell death. Previous reports showed that the MIC values in serum was lower than those in other culture media (Loose et al., 2020). Therefore, the enhanced antibacterial effect in the presence of serum is likely attributed to the combination effect of colistin and depolymerase on the bacterial cell surface facilitating the host immune attack.

Biofilm formation is one of the major contributors for the chronicity of *A. baumannii* infections and their increased antibiotic resistance (Martí et al., 2011). As the EPS can account for 80–90% of the biofilm matrix, the ability of phage in eradicating biofilms was reported to be accounted for the action of their tailspike depolymerases degrading the EPS, facilitating their diffusion through the dispersed biofilms to get access to the underneath bacteria (Dunsing et al., 2019). The effectiveness of recombinant depolymerases in preventing biofilm formation and disrupting the established biofilms has also been studied. The susceptibility of biofilms to phage depolymerase treatments varied, depending on the bacterial strains and the activity of depolymerases. In most reported antibiofilm studies, depolymerases were able to cause a 10–40% biofilm reduction compared with the untreated controls in a dose-dependent manner (Gutiérrez et al., 2015; Hernandez-Morales et al., 2018; Wu et al., 2019; Shahed-Al-Mahmud et al., 2021; Topka-Bielecka et al., 2021). However, there were also reports showing depolymerases were ineffective in dispersing the biofilms, though they were capable of decapsulating bacterial CPS (Latka and Drulis-Kawa, 2020). Overall, the depolymerase treatment were unable to completely inhibit or remove biofilms and the number of viable bacterial counts in the biofilms were similar to the untreated controls (Gutiérrez et al., 2015; Hernandez-Morales et al., 2018; Wu et al., 2019; Topka-Bielecka et al., 2021), with a few exceptions (Bansal et al., 2015; Shahed-Al-Mahmud et al., 2021). These suggested that using depolymerases as a stand-alone treatment might not be sufficient in controlling infections associated with biofilms. Impairing drug diffusion (subdiffusion) within the biofilm matrix is a major contributor to the sub-optimal treatment to biofilm-related infections (Sweeney et al., 2020). Improving the penetration of antibiotics into the biofilm matrix may hold the key to better clinical outcomes. In the present study, Dpo71 demonstrated moderate biofilm inhibition and removal capacities, both around 40% reduction compared with the PBS control, at an optimal concentration of 10 μg/ml (Figures 5, 6). Consistent with most previous studies, Dpo71 could effectively disperse the biofilms but failed to reduce the viable bacterial counts in the biofilms and this was also visually reflected in the LIVE/DEAD confocal images. Combination treatment with colistin, which is the only antibiotic that the MDR-AB2 strain is susceptible to, was studied. The residual biomass and the number of viable bacterial counts within the biofilms were both significantly reduced compared with the depolymerase-alone and colistin-alone treatments, confirming the positive effect of depolymerase-antibiotic combination treatment noted in other bacterial species (Bansal et al., 2014, 2015; Wu et al., 2019). Previously, Dunsing et al. (2019) have proved that treating established *Pantoea stewartii* biofilms with phage tailspike proteins could rapidly restore unhindered diffusion of nanoparticles. Therefore, the improved antibiofilm ability with the combined Dpo71 and colistin treatment noted here was likely attributed to the improved colistin penetration within the biofilm matrix after the EPS depolymerization by the Dpo71. Overall, our data support the depolymerase and antibiotic combination as a promising alternative treatment strategy in managing biofilm-associated infections caused by *A. baumannii*.

The *G. mellonella* infection model was first developed to study the bacterial pathogenicity by Peleg et al. (2009) and has emerged as a valuable inset model to evaluate the effectiveness of novel antibacterial reagents (Yang et al., 2015; Blasco et al., 2020). Several reasons make it a popular model: the larvae (1) can survive at 37°C to mimic the physiological condition of humans; (2) have fast reproduction time to allow high-throughput of experiments compared with mammalian systems; (3) have a semi-complex cellular and humoral innate immunity, which shares remarkable similarities with mammals, but no adaptive immune response to interfere with the therapeutic outcome (Kay et al., 2019). Importantly, the *G. mellonella* model does not require ethical approval to provide informed data in reducing the number of mammals used for further identification/confirmation of the potential lead compounds. Liu et al. (2019b) first evaluated the *in vivo* efficacy of Dpo48 identified from an *A. baumannii* phage (IME200) using a *G. mellonella* infection model. They showed that the Dpo48 treated *G. mellonella* had a higher survival rate (10-30%) than the untreated group at all the time points throughout the study period (72 h). Although the Dpo48 treatment outcome was not particularly profound in the *G. mellonella* infection model, they showed that the Dpo48 could significantly reduce the bacterial load 6 h post-treatment and rescue 100% of the infected mice (both normal and immunocompromised) from fatal sepsis. They attributed the difference in the insect and mammal infection models to the simpler innate immune response of insects. Nonetheless, their results confirmed that the *G. mellonella* infection model is sufficient to predict the antivirulence capacity of depolymerase and their ability to control *A. baumannii* infections in mammals. As shown in Figure 7B, the survival rate of infected *G. mellonella* treated by Dpo71 or colistin monotherapy was around 40%, but the survival rate of those treated by the combination of Dpo71 and colistin could be significantly enhanced to 80%. The results confirmed that Dpo71 was effective in reducing the virulence level of MDR-AB2 *in vivo* and prolonged the survival time of the infected worms as demonstrated in Liu et al. (2019b). Moreover, the adjuvant effect of depolymerase to colistin was demonstrated *in vivo*, consistent with the *in vitro* biofilm experiments. These results warrant further studies on assessing the potential of the combination in treating biofilm-associated infections in mammals.

While promising effects on the use of depolymerase as antivirulence agents have been demonstrated *in vivo*, knowledge on the exact mechanisms of *A. baumannii* CPS cleavage by phage depolymerases are still largely missing (Popova et al., 2020). In addition, depolymerases also present high specificity toward a narrow range of target polysaccharides (specific capsular type of the bacteria) (Blundell-Hunter et al., 2021). In some cases, depolymerases might only be active against a subset of bacteria of their parent phage which are already specific to a small set of bacteria strains (Liu et al., 2020; Topka-Bielecka et al., 2021). Such narrow host spectrum would greatly limit the wider therapeutic application of depolymerases. To address this limitation, the use of cocktails of depolymerases, an approach widely adopted for phage therapy in human use, may be a feasible solution. Further work on elucidating mechanisms of action of depolymerases would allow protein engineering to extend their host range and activity (Latka et al., 2021), facilitating their application as a stand-alone treatment or as an adjuvant with SOC antibiotics.

## Conclusion

In summary, phage tail fiber proteins with depolymerase activity are promising antivirulence agents to re-sensitize *A. baumannii*, even the drug-resistant strains, to host immune attack. The identified Dpo71 depolymerase was found to effectively degrade bacterial capsules with excellent stability at various pH and upon storage. In addition, Dop71 alone can be utilized to prevent and remove *A. baumannii* biofilms. The combination of Dpo71 and colistin was further demonstrated to significantly enhance the antibiofilm activity compared with the monotherapies. Furthermore, this depolymerase was able to enhance the colistin antibacterial activity *in vivo*, markedly improving the survival rate of infected *G. mellonella*. As carbapenem-resistant *A. baumannii* has been ranked as the number one priority pathogen by the WHO and there are no antibiotics which have reached the advanced stage in the development pipeline to target this superbug, depolymerases as a stand-alone treatment or adjuvant to antibiotics may represent promising treatment strategies in controlling multidrug-resistant *A. baumannii* infections.

## Data Availability Statement

The datasets presented in this study can be found in online repositories. The names of the repository/repositories and accession number(s) can be found below: https://www.ncbi.nlm.nih.gov/genbank/, JX976549.

## Author Contributions

XC was responsible for data collection and interpretation for the overall study and manuscript writing. ML was responsible for data collection and interpretation for the depolymerase characterization. PZ was responsible for data collection and interpretation for the *Galleria mellonella* infection model. MX and WY were responsible for the SEM and confocal imaging experiments. LB, YL, and JX were responsible for the conceptual design of the study, data interpretation, and manuscript review. SL was responsible for the coordination of the study, conceptual design, data interpretation, and manuscript review. All authors contributed to the article and approved the submitted version.

## Conflict of Interest

The authors declare that the research was conducted in the absence of any commercial or financial relationships that could be construed as a potential conflict of interest.

## Publisher’s Note

All claims expressed in this article are solely those of the authors and do not necessarily represent those of their affiliated organizations, or those of the publisher, the editors and the reviewers. Any product that may be evaluated in this article, or claim that may be made by its manufacturer, is not guaranteed or endorsed by the publisher.
