# Supplementary material for: Phage-Derived Depolymerase as an Antibiotic Adjuvant Against Multidrug-Resistant Acinetobacter baumannii
Source: Front Microbiol. 2022 Mar 25;13:845500. doi: 10.3389/fmicb.2022.845500 (PMC8990738; doi:10.3389/fmicb.2022.845500)
Supplement: Supplementary file 1 [file Data_Sheet_1.docx]

Supporting Information

**Phage-Derived Depolymerase as an Antibiotic Adjuvant Against Multidrug-Resistant *Acinetobacter Baumannii***

Xi Chen^1^, Miao Liu^1^, Pengfei Zhang^2^, Miao Xu^2^, Weihao Yuan^3^, Liming Bian^3^, Yannan Liu^4,*^, Jiang Xia^1, *^, Sharon S.Y. Leung^2, *^

^1^Department of Chemistry, The Chinese University of Hong Kong, Hong Kong SAR, China

^2^School of Pharmacy, The Chinese University of Hong Kong, Hong Kong SAR, China

^3^Department of Biomedical Engineering, The Chinese University of Hong Kong, Hong Kong SAR, China

^4^Emergency Medicine Clinical Research Center, Beijing Chao-Yang Hospital, Capital Medical University, Beijing, 100020, China

*To whom correspondence may be addressed. Email: Sharon Shui Yee Leung, [sharon.leung@cuhk.edu.hk](mailto:sharon.leung@cuhk.edu.hk), Jiang Xia, [jiangxia@cuhk.edu.hk](mailto:jiangxia@cuhk.edu.hk) and Yannan Liu, yannan_liu@foxmail.com.

**Contents**

| Items | Pages |
| --- | --- |
| **Table S1**. Bacterial strains used in this study. | S3 |
| **Table S2**. Protein sequence of Dpo71. | S3 |
| **Table S3**. MIC of colistin against *A. baumannii.* | S3 |
| **Table S4**. Bacterial resistance table of depolymerase Dpo71 against *A. baumannii.* | S4 |
| **Figure S1**. Capsule depolymerase activity of Dpo71. | S5 |
| **Figure S2**. Time-killing curve for Dpo71against AB#1 strain in human serum. | S6 |
| **Figure S3**. Representative spot test images for resistance assay. | S7 |

**Table S1**. Bacterial strains used in this study and activity spectrum of depolymerase against *A. baumannii.* (**+** indicating sensitive; **-** indicating insensitive).

Two A. baumannii strains (AB#1 and AB#2) sensitive to the parent IME-AB2 phage and other two are insensitive (AB#3 and AB#4) strains.

**Table S2**. Protein sequence of Dpo71

Protein id: YP_009592222.1

MGSSHHHHHHSSGLVPRGSHMASMTGGQQMGRGSMTNPTLITTPFAENGDKNTIPESVGANPQNATMQAGFPPITQQKISEGGIPPERNDFNGILNLYGQHIVHLNKGLPYEFDQAFANAIGGYPLNARLMLDNGDIVKSTVPNNTNNPNSDMTGWVKINSASQIFDESGLSQQEINNGVKSAAALRLLNPNGEGERIYLISFNEGQGEGGGVFISKNKGTLVDDGGTILQSSNASIVYVRINFDSLTPEMFGAKGDDISFDNYLALQAAFKHPLPLEIPPKTYYTTRSIWYTSGKKIKGSGHQKSVICKTTNSTETDLPSSFAKDAVIIARHWSANDYAHYCEFDGFLVTSTVKCEFGLYAPRIAESSFSNFKIHNAIKGFYSDDAWMISMIRVTSSSDRPYIVNMGTSITMNSCWAINARGESSYCYEFNNLYYSTLISCGADNNGLDGSPIKALYRVLNSSITFISCASENNHAYKIFHGQGASVTIVKMTAMRFYNKYKVSNPVWGDENALFDLRSETRLDIKDSAFDGLLNTDTSTSPSWINITDTSYLNYSNVRVNIPITGASVNDTTAFGVKWGSVTTVILDFGTYKIESCVNLPSNLYNPLSTPTNETYSSIFTRGSIRENGRELSTENLNNLRYLGTITFKQSQSAEATLANNYPENGGAWVIQQYSDVATDSSNVNTNTTQIAIRKDSNRIYFRCAPYGGIFTPWYKIYHSGNTTVDANGFLKAIEF*

**Table S3**. MIC value of colistin against *A. baumannii*. (MIC is 2µg/ml)

**Table S4**. Bacterial resistance table of depolymerase Dpo71 against *A. baumannii* (+ indicating sensitive; - indicating insensitive).


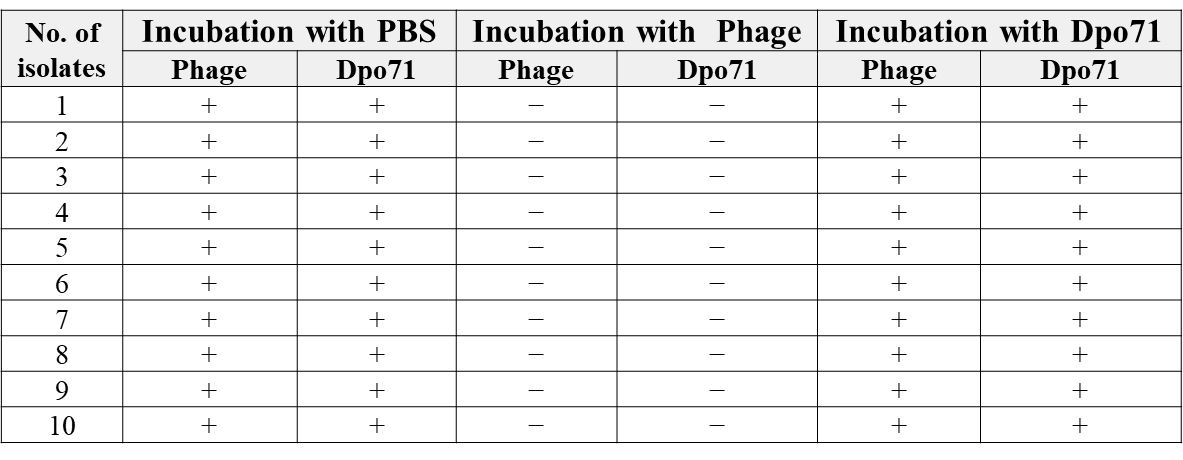


One of the limitations of phage therapy is known to be the development of resistance. So, we evaluated whether Dpo71 can induce resistance development. The MDR-AB2 strain (10^6^ CFU/ml) was challenged with the parent IME-AB2 phage (MOI of 10), Dpo71 (100 μg/ml, final concentration) or PBS for 24 h (37 °C, 120 rpm) and tested for the emergence of resistant phenotypes (**Table S3**). The incubated culture were subcultured three times in agar plates to guarantee that the colonies were free of phage and Dpo71. Ten random bacterial colonies of each culture were selected to estimate the sensitivity to the phage or Dpo71 using the spot test assay. Representative spot test figures are shown in **Figure S2**. Challenged bacteria were considered resistant to phage or Dpo71 when no plaque/inhibition halo was observed. PBS treated bacteria all remained sensitive to both the IME-AB2 phage and Dpo71. Pre-challenged with IME-AB2 phage resulted in resistance to both the phage and Dpo71 afterwards. Although Dpo71 was originated from the same phage, all ten bacterial colonies tested remained susceptible to the enzyme without resistance development. This observation also agrees with the findings reported by Oliveira et al. (2019). These data, although preliminary, show that Dpo71 as a therapeutic option is less likely to induce resistance than the parent phage.


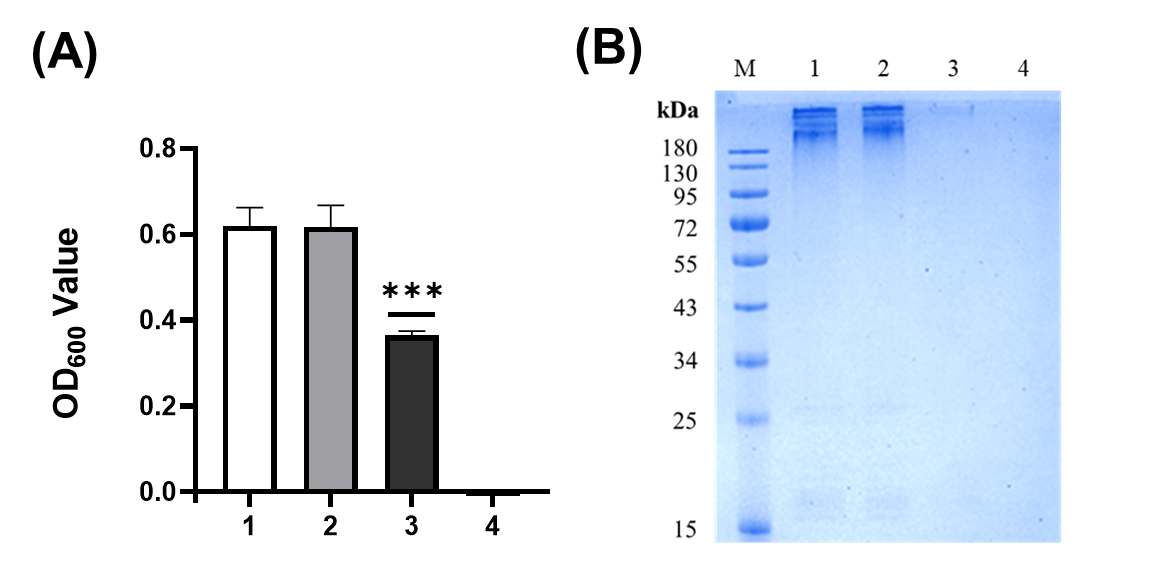


**Figure S1** Capsule depolymerase activity of Dpo71. (A) Polysaccharide substrate (CPS/LPS) extracted from *A. baumannii* was treated with Dpo71 at 37°C for 2 h, the cetylpyridinium chloride (CPC) was added and incubated for 5 min at RT. The turbidity of residual polysaccharide extracts (CPS/LPS) was measured at 600 nm. Data are presented as means ± SD (n = 4, ***P < 0.001, Student’s t-test.). (B) Reaction mixtures were separated by 10% SDS-PAGE, followed by Alcian blue staining. 1: polysaccharide extracts (CPS/LPS) only; 2: polysaccharide extracts + heat inactivate Dpo71; 3: polysaccharide extracts + Dpo71; 4: Dpo71 only; M: marker.

The polysaccharide extracts (CPS/LPS) degradation activity of Dpo71 was evaluated by monitoring the turbidity of the residual polysaccharide extracts. Upon incubation with Dpo71, the OD_600_ value decreased from 0.6 to 0.3, while the heat inactive enzymes did not decrease the turbidity and the enzyme itself had no influence on the turbidity (**Figure S1 A**). The reduced OD_600_ value of the Dpo71 treated polysaccharide extracts suggesting the polysaccharide degradation capacity of Dpo71. Its depolymerase activity were further verified by gel electrophoresis followed by Alcian blue staining as previously described (Liu et al., 2020). The treated samples were separated by a 10% SDS-PAGE. The gel was then washed with the fix/wash solution (25% ethanol, 10% acetic acid in water) and stained by 0.1% Alcian blue (Sigma-Aldrich) dissolved in the fix/wash solution for 15 min in the dark. Finally the polysaccharide extracts were visualized blue after the gel was destained overnight in the fix/wash solution. **Figure S1 B** confirms the degradation of the polysaccharide extracts.


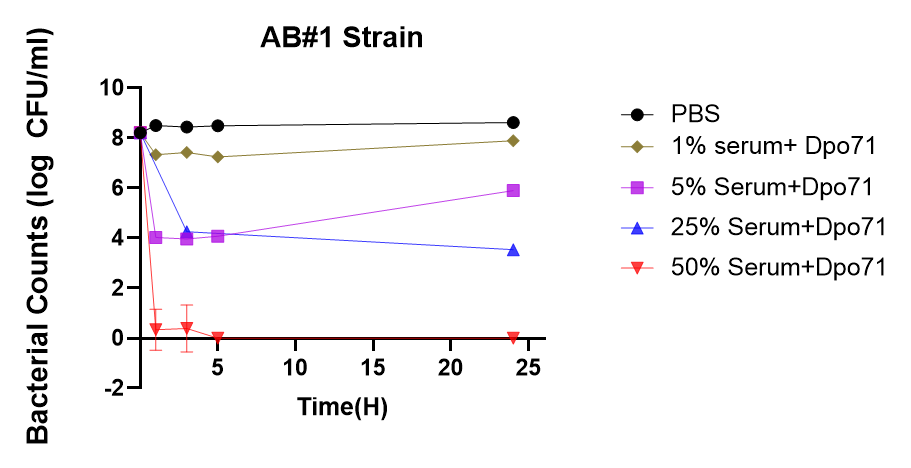


**Figure S2** Time-killing curve for Dpo71 against AB#1 strain in human serum, human serum ranging from 1% to 50% (volume ratio) and Dpo71 concentration of 10 µg/ml. Data are expressed as means ± SD (n = 3).

The time killing assay on the sensitive strain (AB#1) was performed with a serum volume ratio of 1% to 50%. Similar as the other sensitive strain (AB#2), the result of antibacterial activity was shown on a serum ratio-dependent manner. Complete bacterial eradication was also observed after 5 h treatment at 50% human serum for AB#1. A 5% serum was sufficient to achieve around 4-log bacterial reduction after 5 h treatment and with partial regrowth after 24 h.


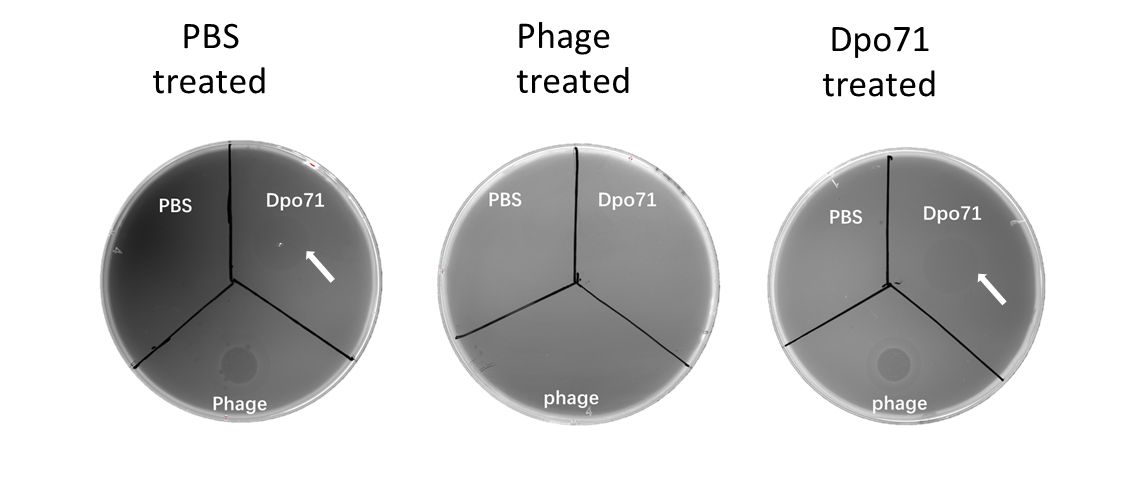


**Figure S3** Bacterial resistance development of Dpo71. Dpo71 and IME-AB2 phage. A. baumannii cells were incubated with PBS buffer, IME-AB2 phage, or Dpo71 and afterwards tested for their activity against the phage or depolymerase using the spot tests. Representative spot test figures for treated *A. baumannii.*

For the PBS and depolymerase treated *A. baumannii*, the semi-clear spot formation indicated that the Dpo71 was still active against the challenged strain. And the clear plaque can be detected from the PBS and depolymerase treated group, indicating that these two groups of bacteria remained susceptible to the phage without resistance development. However, no inhibition halo was observed for both the Dpo71 and IME-AB2 phage spot, for the group challenged with the phage, suggesting the resistance to both the phage and Dpo71.

**REFERENCES**

Liu Y, Leung SSY, Huang Y, Guo Y, Jiang N, Li P, Chen J, Wang R, Bai C, Mi Z, Gao Z. 2020. Identification of two depolymerases from phage IME205 and their antivirulent functions on K47 capsule of *Klebsiella pneumoniae*. Front Microbiol 11:218.

Oliveira H, Mendes A, Fraga AG, Ferreira A, Pimenta AI, Mil-Homens D, Fialho AM, Pedrosa J, Azeredo J. 2019. K2 capsule depolymerase is highly stable, is refractory to resistance, and protects larvae and mice from *Acinetobacter baumannii* sepsis. Appl Environ Microbiol 85(17):e00934-19.
